# IUC23534-82 Incidental detection of renal masses following implementation of abdominal imaging in prostate mpMRI protocol

**DOI:** 10.1093/oncolo/oyaf248.004

**Published:** 2025-08-29

**Authors:** F R Antara, M Hussain, S Tadtayev, A Chapman

**Affiliations:** Ashford and St Peters Hospitals NHS Foundation Trusts, United Kingdom; Ashford and St Peters Hospitals NHS Foundation Trusts, United Kingdom; Ashford and St Peters Hospitals NHS Foundation Trusts, United Kingdom; Ashford and St Peters Hospitals NHS Foundation Trusts, United Kingdom

## Abstract

**Background:**

Multiparametric MRI (mpMRI) of the prostate has become integral in the diagnosis and management of prostate cancer. Traditionally limited to the pelvis, recent protocol changes at Ashford and St Peter’s Hospitals (ASPH) from July 4, 2023 have included T2-weighted abdominal sequences to aid complete staging and to align the protocol to one used at Royal Surrey County Hospital (RSCH). This study assesses the impact of this addition on the incidental detection of renal masses.

**Methods:**

A retrospective analysis was conducted of all mpMRI prostate scans performed at ASPH between July 4, 2023 and July 3, 2024. Exclusion criteria included repeat or incomplete scans. Renal findings were reviewed and categorized, and further imaging or interventions were recorded.

**Results:**

Out of 1,004 scans, 999 met the inclusion criteria. Only 2% of cases required additional imaging to clarify renal findings. The addition of abdominal imaging led to the detection of renal abnormalities in several cases, including 6 solid renal masses and one Bosniak IIf cyst later upgraded (0.7% cases). Among these patients, 4 either underwent or are awaiting partial or radical nephrectomy, and 4 are being managed with active surveillance. The estimated scan time increase was approximately 11.6 hours per actionable lesion detected, totaling 116 extra scanner hours.

**Conclusions:**

Incorporation of abdominal sequences into prostate mpMRI protocols has led to a diagnostic renal yield of 0.7%, surpassing that seen in some screening modalities. Although there has been an increase in scanning time and reporting workload, these incidental renal findings may contribute meaningfully to early cancer detection in addition to a more complete prostate staging provided by the new protocol.

